# Competition between the tadpoles of Japanese toads *versus* frogs

**DOI:** 10.1038/s41598-022-05525-z

**Published:** 2022-01-31

**Authors:** Takashi Haramura, Koshiro Eto, Michael R. Crossland, Kanto Nishikawa, Richard Shine

**Affiliations:** 1 Department of Environmental Sciences, Rakuno Gakuen University, Hokkaido, Japan; 2 Graduate School of Human and Environmental Studies, Kyoto University, Kyoto, Japan; 3 Kitakyushu Museum of Natural History & Human History, Kitakyushu, Fukuoka Japan; 4 School of Life and Environmental Sciences, University of Sydney, Camperdown, NSW 2006 Australia; 5 Graduate School of Global Environmental Studies, Kyoto University, Kyoto, Japan; 6 School of Natural Sciences, Macquarie University, North Ryde, NSW 2109 Australia

**Keywords:** Evolutionary ecology, Coevolution

## Abstract

Competition within and among species can play a key role in structuring the assemblages of anuran tadpoles. Previous studies have reported that tadpoles of the invasive cane toad (*Rhinella marina*) are more strongly disadvantaged by the presence of native frog tadpoles than by the same number of conspecific toad tadpoles. That effect might arise from a lack of coevolution of the invasive toad with its competitors; and/or from a generalized superiority of frog tadpoles over toad tadpoles. To clarify those possibilities, we conducted experimental trials using the larvae of a native rather than invasive toad (*Bufo japonicus formosus* in Japan) exposed to larvae of native anurans (the sympatric frogs *Rana japonica* and *Rana ornativentris* and the parapatric toad *Bufo japonicus japonicus*). In intraspecific competition trials, higher densities of *B. j. formosus* prolonged the larval period and reduced size at metamorphosis, but did not affect survival. In interspecific competition trials, the effects of the other anuran species on *B. j. formosus* were similar to the effects of the same number of conspecific larvae. This similarity in impact of interspecific *versus* intraspecific competition argues against any overall competitive superiority of frog larvae over toad larvae. Instead, the vulnerability of larval cane toads to frog tadpoles may result from a lack of coevolutionary history.

## Introduction

Many ecosystems contain a diverse array of species that overlap considerably in the resources upon which they rely; and ecological theory suggests that such overlap may lead to intense competition, that in turn may favour adaptations to reduce niche overlap^1–3^. The larvae of anuran amphibians (tadpoles) have been “model organisms” for experimental studies of competitive effects, because a single waterbody often contains larvae of multiple taxa with high niche overlap, that compete for finite food resources^4–6^. Extensive studies have confirmed that intra- and interspecific competition can affect larval survival rates, larval periods, and body mass at metamorphosis^6–13^, and lead to the evolution of mechanisms for suppression of competing larvae (e.g.^14,15^).

One interesting example of competitive suppression involves the invasive cane toad (*Rhinella marina*). Experimental studies in two parts of the toads' invasive range (Australia and Ishigaki Island, Japan) have shown that the survival and growth of larval cane toads is strongly reduced by the presence of frog tadpoles^16–19^, via exploitative competition for food^20^. In these studies, the viability of a cane toad tadpole was reduced more by competition with a frog tadpole than with a conspecific toad tadpole—but why? Possible answers include a generalised competitive superiority of frog larvae over toad larvae, perhaps because the former are often larger than the latter; and/or an effect of coevolution, whereby cane toads are highly vulnerable because they have encountered these frog species only recently (for decades, at most) and hence have not yet evolved mechanisms to reduce that vulnerability.

To explore this question, we can examine the effects of intraspecific and interspecific competition on a toad species that is native rather than invasive—and hence, has had the opportunity to adapt to sympatric frogs over evolutionary time. We can also examine the sensitivity of such a toad to competition from closely related bufonid species that are parapatric to the target taxon, to compare competitive impacts of toads *versus* frogs. No such studies were possible in earlier studies of cane toads in Australia and on Ishigaki Island (Okinawa, Japan) because neither of those areas contains native toads.

We have conducted such a study using a toad taxon that is native to Japan (*Bufo japonicus formosus)* as our target species, two ranid frogs as sympatric competitors (*Rana japonica, Rana ornativentris*) and a closely related subspecies as the parapatric toad competitor (*Bufo japonicus japonicus*)*.* All four species breed at the same time of year, such that the tadpoles of *B. j. formosus* co-occur with the two *Rana* species in temporary waterbodies. We manipulated the numbers and identity of tadpoles in experimental containers to (1) quantify the effects of intraspecific competition, by raising tadpoles of *B. j. japonicus* at a range of densities; and (2) quantify the effects of interspecific competition, by raising tadpoles at a fixed total larval density but changing the composition of the assemblage in terms of the relative numbers of toad *versus* frog competitors.

## Methods

### Study species

Our laboratory studies included four anuran taxa, all of which are terrestrial and breed in wide array of freshwater habitats including ponds, marshes and swamps. Two species belong to the “true toads” (family Bufonidae). Our target species was the eastern-Japanese common toad (*Bufo japonicus formosus*; total length [= TL] of tadpoles up to 30 mm^21^), and the parapatric bufonid was the western-Japanese common toad (*Bufo japonicus japonicus*; tadpole TL generally up to 35 mm^21^). The other two taxa are members of the family Ranidae, both of which are broadly sympatric with *B. j. formosus*: the Japanese brown frog (*Rana japonica*; mean tadpole TL = 38 mm^21^) and the montane brown frog (*Rana ornativentris*; mean tadpole TL = 43 mm^21^). Larval body sizes in the two groups used in the experiment spanned a similar range (Table 1).

**Table 1:** Body sizes (mean ± standard errors, and range) and Gosner stages for tadpoles as measured at the beginning of the experiments.

	Body size (mm)	Mass (g)	Developmental stage^33^
*Bufo japonicus formosus*	6.17 ± 0.13 (5.27–7.43)	0.028 ± 0.001 (0.016–0.037)	26.5 ± 0.35 (25–29)
*Bufo japonicus japonicus*	7.26 ± 0.13 (6.50–8.03)	0.054 ± 0.002 (0.050–0.066)	27.9 ± 0.51 (26–31)
*Rana japonica*	5.62 ± 0.13 (4.54–6.12)	0.025 ± 0.002 (0.014–0.035)	26.3 ± 0.30 (25–28)
*Rana ornativentris*	6.57 ± 0.19 (5.74–7.69)	0.046 ± 0.004 (0.031–0.067)	25.8 ± 0.49 (25–30)

Tadpoles of all four species were derived from eggs collected in natural waterbodies from two sites (*B. j. formosus* and *R. japonica*—Tochigi prefecture, *B. j. japonicus* and *R. ornativentris*—Okayama prefecture) during the period 15–31 March 2014. Tadpoles of *B. j. formosus*, *R. japonica* and *R. ornativentris* were found in the same waterbodies (Haramura, personal observation), confirming that competition is likely to occur in nature. To equalize developmental stage of tadpoles at the onset of the experiment as much as possible, eggs or embryos of early-laid clutches were kept in cool conditions (12 °C) prior to the main experiment. Tadpoles of all four species were maintained in groups in 120 L plastic containers (66 × 86 × 34 cm). Tadpoles were fed algal pellets (Hikari Algae Wafers, Kyorin) ad libitum, and water was changed weekly. The tadpoles used in the experiment were haphazardly selected from these containers and added to experimental bins as described below.

### Laboratory experiments

Experiments were conducted using plastic tanks (26 × 38 × 23 cm), each filled with 23 L water and located in a covered building exposed to ambient temperatures. At the start of the experiment, we added a 2 cm layer of soil substrate and 3 g of algal pellets to each bin. We did not provide additional food for the remainder of the experiment. Tadpoles varied in sizes and developmental stages at the beginning of the experiment (see Table 1).

Our experiment consisted of six treatments: (1) 5 larvae of *B. j. formosus*, (2) 15 *B. j. formosus*, (3) 50 *B. j. formosus*, (4) 25 *B. j. formosus* plus 25 *B. j. japonicus*, (5) 25 *B. j. formosus* plus 25 *R. japonica*, and (6) 25 *B. j. formosus* plus 25 *R. ornativentris*. The experiment was a complete randomised block design, with 5 replicate tanks per treatment. We recorded the number of *B. j. formosus* to metamorphose from each tank (survival), as well as the larval period, and length (snout to urostyle length = SUL) and mass of each *B. j. formosus* metamorph from each tank. Treatments 1, 2 and 3 allowed us to assess the effect of intraspecific competition on *B. j. formosus*, whereas treatments 3, 4, 5 and 6 allowed us to assess the strength of interspecific *versus* intraspecific competition at standardised density. We also measured water temperature and pH in each tank every 4 days.

Because it is not possible to visually distinguish between metamorphs of *B. j. formosus* and *B. j. japonicus*, we used a Loop-Mediated Isothermal Amplification (LAMP) assay to distinguish between these two subspecies in the interspecific competition experiments. LAMP is a genetic method which detects the presence/absence of a specific DNA sequence in the tested sample^22^. Total DNA of each metamorph was extracted from frozen tissue using the DNeasy Blood and Tissue Kit (QIAGEN) with standard protocols. Following extraction, each sample was tested by LAMP assay in two independent systems—assays with *B. j. japonicus*-positive and *B. j. formosus*-positive primer sets. For the primer set used and the experimental conditions, we followed the methods with slight modification^23^. The reaction mixtures were incubated at 63–65 °C for 90 min and then heated at 95 °C for 2 min to terminate the reaction.

### Statistical analyses

We analysed treatment effects on both water temperature and pH using ANOVA. We analysed treatment effects on larval period, metamorph SUL and metamorph mass using linear models (MANOVA, followed by ANOVA), with treatment and spatial block as fixed effects (JMP 9.0, SAS Institute, Cary, NC, USA). MANOVA analyses were based on tank means to avoid pseudoreplication (the JMP statistical package does not support MANOVA with random effects). ANOVA analyses were based on data for all individuals per tank, using tank as a random effect. When the overall ANOVA gave a significant result, we performed post hoc Tukey’s HSD tests for pairwise comparison of treatments. We analysed survival to metamorphosis as a binomial response (alive, dead^24^) using ANOVA, with treatment and spatial block as fixed effects (package carData^25,26^). Survival analyses were based on the quasi-binomial distribution to account for overdispersion of data. Alpha level was set at p = 0.05 in all analyses.

### Ethics approval

All procedures were approved by Rakuno Gakuen University Animal Care Committee (permit #DH21D6). The study was carried out in compliance with the ARRIVE guidelines, and all methods were carried out in accordance with relevant guidelines and regulations.

## Results

The average water temperature and pH in tanks was 19.29 ± 0.10 °C (SE, range: 17.0–22.5) and 8.59 ± 0.01 (SE, range 8.2–8.9) respectively. There was no significant difference among treatments (water temperature: F = 0.0086, df = 5, p = 1.0000, pH: F = 0.0063, df = 5, p = 1.0000).

### Intraspecific competition (density = 5, 15, 50 tadpoles per tank)

**Figure 1:**
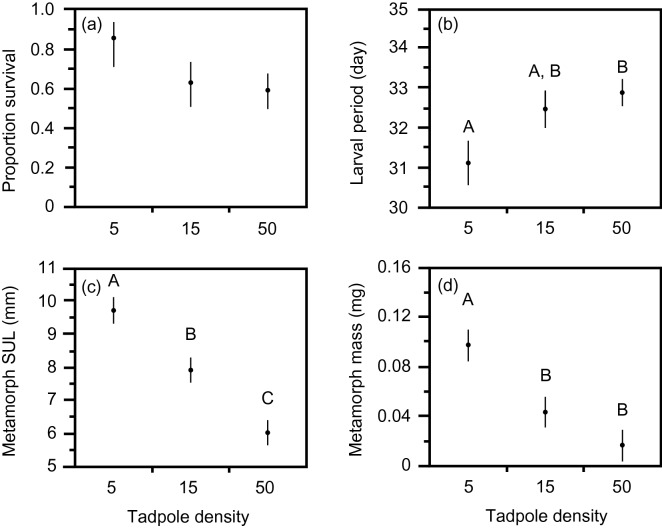
The effect of tadpole density on survival, larval period and metamorph size of toads, *Bufo japonicus formosus*. The treatments comprised densities of 5, 15 or 50 tadpoles per container. The panels show impacts on (**a**) survival rate, (**b**) larval period, (**c**) metamorph snout-urostyle length, and (**d**) metamorph mass. The graphs show mean values (based on 5 replicate containers per treatment) with standard errors. The same letter indicates that the differences are not significant using a post hoc test (Tukey’s HSD) at the 0.05 level.

The density of conspecifics did not have any significant effect on survival to metamorphosis of *B. j. formosus* (treatment: Wald chi-square = 3.468, df = 2, p = 0.1766; block: Wald chi-square = 7.770, df = 4, p = 0.1004; Fig. 1a). However, conspecific density had a significant effect on the combined responses of variables (larval period, metamorph SUL, metamorph mass) of *B. j. formosus* (MANOVA treatment: Wilks’ Lambda = 0.0181, F = 10.7224, df = 6, 10, p = 0.0007; block: Wilks’ Lambda = 0.2028, F = 0.9326, df = 12, 13.52, p = 0.5441). Higher densities of conspecifics increased the duration of the larval period (treatment: F = 6.678, df = 2, 9.30, p = 0.0159; block: F = 0.817, df = 4, 0.40, p = 0.7574; Fig. 1b), and decreased size at metamorphosis (SUL—treatment: F = 49.729, df = 2, 6.94, p < 0.0001; block: F = 1.154, df = 4, 6.88, p = 0.4074; Fig. 1c; mass—treatment: F = 22.949, df = 2, 6.66, p = 0.0010; block: F = 1.031, df = 4, 6.68, p = 0.4566; Fig. 1 d).

### Interspecific competition (density = 50 tadpoles per tank)

**Figure 2:**
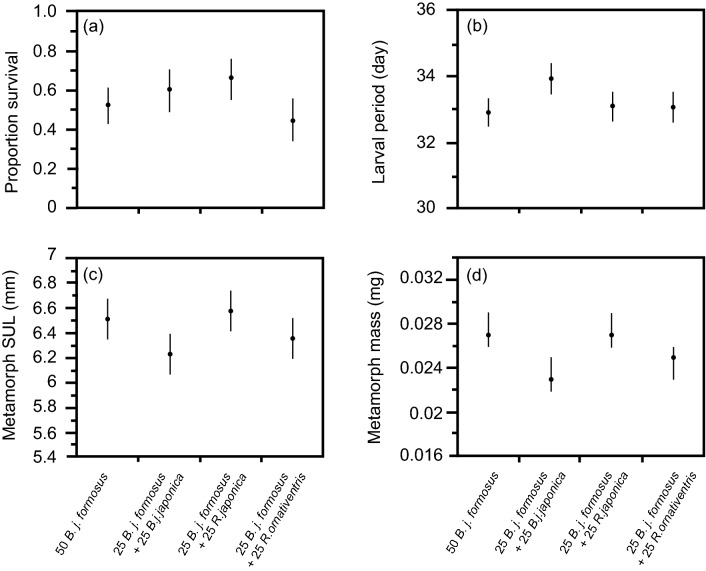
The effect of interspecific competition on survival, larval period and metamorph size of toads, *Bufo japonicus formosus.* The treatments comprised 50 *B. j. formosus* tadpoles, or 25 tadpoles of *B. j. formosus* plus 25 tadpoles of *B. j. japonicus*, *Rana japonica* or *R. ornativentris*. The panels show impacts on (**a**) survival rate, (**b**) larval period, (**c**) metamorph snout-urostyle length, and (**d**) metamorph mass. The graphs show mean values (based on 5 replicate containers per treatment) with standard errors.

There was no significant effect of treatment (competitor species) on survival of *B. j. formosus* to metamorphosis (treatment: Wald chi-square = 4.076, df = 3, p = 0.2533; block: Wald chi-square = 2.708, df = 4, p = 0.6078; Fig. 2a). MANOVA also showed no significant effect of treatment on overall responses (i.e., including variables of larval period, metamorph SUL, metamorph mass) of *B. j. formosus* (treatment: Wilks’ Lambda = 0.3722, F = 1.2275, df = 9, 22.05, p = 0.3285; block: Wilks’ Lambda = 0.3269, F = 1.0565, df = 12, 24.10, p = 0.4344). Although tadpoles of *B. j. japonicus* tended to impose stronger negative effects on *B. j. formosus* than did *B. j. formosus* on itself (Fig. 2b–d), there was also no significant effect of competitor species on the duration of the larval period for *B. j. formosus* (treatment: F = 2.262, df = 3, 9.83, p = 0.1448; block F = 0.783, df = 4, 9.56, p = 0.5627, Fig. 2b), or size at metamorphosis (SUL—treatment: F = 1.895, df = 3, 10.46, p = 0.1917; block: F = 2.039, df = 4, 10.46, p = 0.1615; Fig. 2c; mass—treatment: F = 2.706, df = 3, 10.69, p = 0.0980; block: F = 0.495, df = 4, 10.68, p = 0.7403; Fig. 2d).

## Discussion

In our laboratory experiment, tadpoles of the eastern-Japanese common toad (*Bufo japonicus formosus*) showed strong intraspecific competitive effects: an increase in the number of toad tadpoles per container generated a substantial reduction in rates of growth and development, and in size at metamorphosis (Fig. 1). The main result from interspecific competition treatments, however, was that these effects did not vary among competitors that were either conspecifics or heterospecifics (Fig. 2). That is, the impacts of frog tadpoles on *B. j. formosus* were similar to those of the same number of toad tadpoles (Fig. 2).

Our results accord with previous studies that have shown negative consequences for tadpoles raised at high densities (e.g.^7,27–29^). However, we did not find a stronger competitive effect of frog tadpoles than of toad tadpoles, unlike the results of studies on invasive cane toads in Australia and Okinawa^16–19^. Why, then, are tadpoles of the cane toad more sensitive to the presence of frog tadpoles than conspecific toad tadpoles? At least under the conditions under which we conducted our experiments, the answer does not involve a competitive superiority of frog tadpoles over toad tadpoles: we saw no such effect in our trials (Fig. 2). Instead, the results for cane toads may reflect two aspects of this system. First, most of the Australian frog tadpoles tested were much larger than the toad tadpoles—in some cases, by a 20-fold margin^16–18^. Larger tadpoles may (in general) be better competitors (e.g.^30^), and this effect may be stronger if the size disparity is greater. However, we note that the competitive superiority of native frog tadpoles over invasive cane toads on Ishigaki occurred despite a relatively small difference in body size (native tadpoles up to 1.44 times the size of cane toad tadpoles^19^). In the present study, interspecific differences in tadpole size (Table 1) did not translate to differential competitive effects (Fig. 2), although size differences were relatively minor. Thus, at least over the size range studied in Ishigaki and in the current study, size effects seem unlikely to explain differential vulnerability of cane toad larvae to frog tadpoles than to conspecifics. And as well as body size, outcomes of competition also may be affected by habitat use. However, the tadpoles of all four species used in the present study are primarily bottom-dwellers, minimizing any effects of differential habitat use among species.

The second aspect of the cane toad system is that this is an invasive species; and hence, these toads have had only a brief window of opportunity to adapt in ways that buffer them against the competitive effects of native anurans. In contrast, the toad species that we targeted in the present study (*B. j. formosus*) is sympatric with the frogs we studied (*R. japonica* *R. ornativentris*) over a broad area, and thus likely has coevolved with those frogs over a long period^31,32^. These taxa frequently breed in the same waterbodies, at the same time of year, and thus compete with each other in nature as well as in our laboratory studies. That situation, continuing over long periods, should enable coevolution between the competing taxa, in ways that reduce the negative impacts of competitors. Interestingly, we found no significant competitive effects of parapatric *B. j. japonicus* on *B. j. formosus*. This result may be due to the close phylogenetic relationship (i.e., belonging to the same genus), and thus ecological similarity, between these two species.

Future work could usefully examine competitive interactions between adult anurans as well as between larvae; and could assess the impacts of a broader range of species under a wider range of conditions (including, outdoor enclosures that more accurately mimic spawning sites in nature). In particular, it would be of great interest to examine larval competition within the native range of the cane toad. If this species’ vulnerability to competition from frog tadpoles results from lack of coevolution in invaded areas, then we expect that trials with the tadpoles of South American frog species would provide different results to those seen in Australia and on Ishigaki. That is, cane toad tadpoles should be resilient to the presence of larvae from sympatric frog species, as seen in our work with *B. j. formosus*. More generally, it would be instructive to compare ecological interactions between invasive species and other fauna not only in the areas they have invaded, but also within their native range, to clarify the impacts of translocation on the intensity of interspecific competition.
